# Introduction to the Thematic Review Series: Seeing 2020: lipids and lipid-soluble molecules in the eye

**DOI:** 10.1016/j.jlr.2020.100007

**Published:** 2020-12-09

**Authors:** Steven J. Fliesler

**Affiliations:** Research Service, VA Western New York Healthcare System, Buffalo, NY, USA; Departments of Ophthalmology and Biochemistry and Neuroscience Graduate Program, Jacobs School of Medicine and Biomedical Sciences, SUNY–University at Buffalo, Buffalo, NY, USA

In 2010, this journal published a series of review articles in a Thematic Issue entitled “Lipids and Lipid Metabolism in the Eye.” Over the ensuing decade, a number of significant advances have been made that are pertinent to this broad topic, which prompted us to launch a follow-up Thematic Issue to present updates on several of the topics reviewed in that prior issue as well as to expand into new areas that previously had not been addressed. In addition to considering the conventional classes of lipids (e.g., glycerophospholipids, sphingolipids, fatty acids, and sterols), we also wanted to address some key lipid-soluble molecules (e.g., retinoids, bisretinoids, and carotenoids) that play important physiological roles in ocular tissues. Given that this issue concerns the eye (where normal visual acuity is referred to as “20/20” vision) as well as the fact that we have just emerged from the year 2020, we have purposely employed a bit of double-entendre in naming this Thematic Issue “Seeing 2020: lipids and lipid-soluble molecules in the eye.” Below, we present a brief overview of each of the articles included in this Thematic Issue, proceeding anatomically from the back to the front of the eye. While the majority of these articles concern various aspects of lipid biochemistry and eye diseases pertinent to the retina, we’ve also included articles germane to the lens and cornea (Fig. 1).

**Fig. 1:**
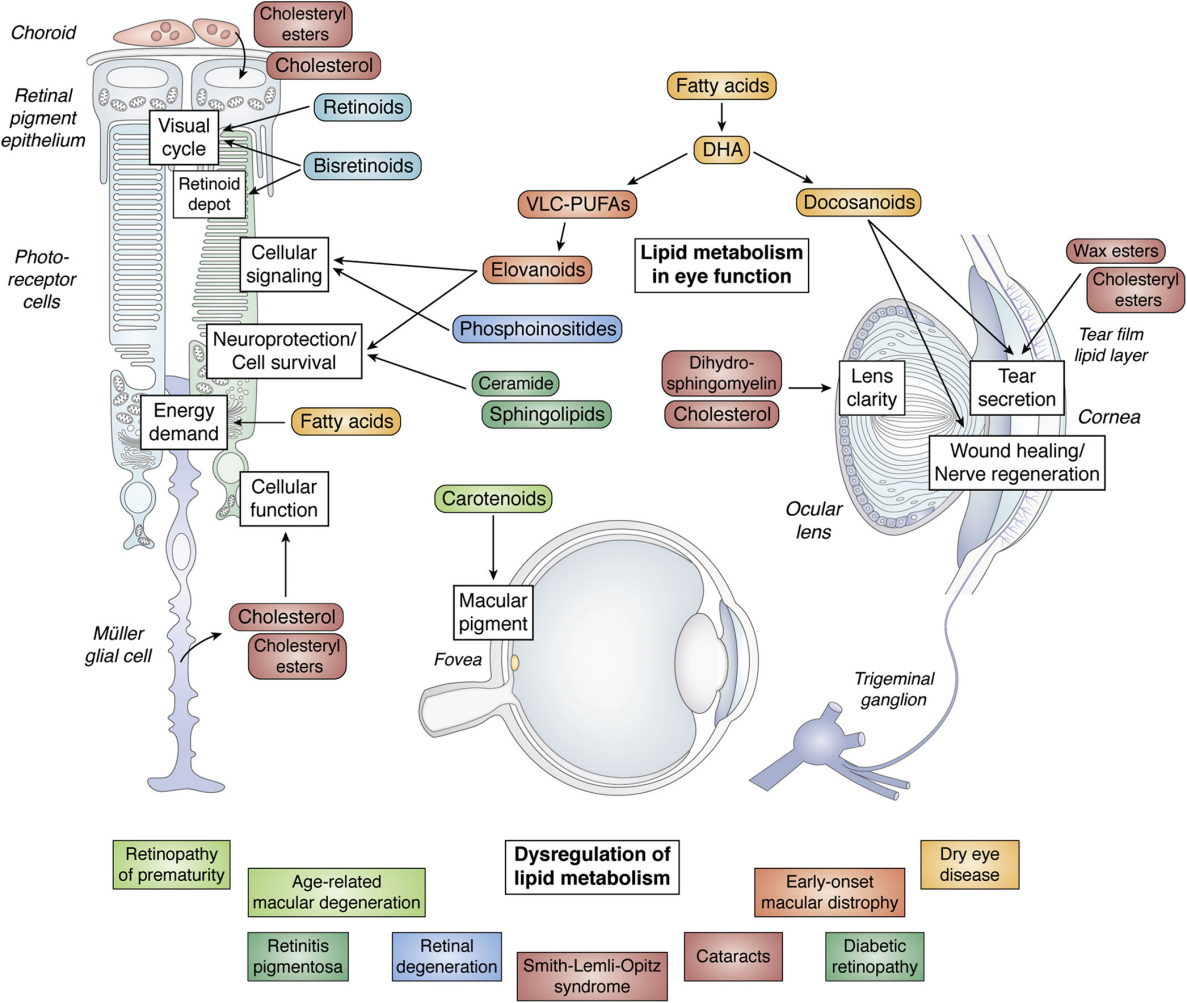
Schematic overview of topics addressed in this Thematic Issue. Artistic credit: Luciana E. Giono, PhD; IFIBYNE UBA CONICET.

Retinoids comprise a diverse family of organic compounds derived from vitamin A that play a central role in the physiology of the retina. Most notably, the 11-*cis* isomer of vitamin A aldehyde (11-*cis* retinaldehyde) serves as the chromophore covalently linked to proteins (rod and cone opsins) to generate the visual pigments of rod and cone photoreceptor cells. The “visual cycle” involves a series of events, including conversion of vitamin A alcohol (all-*trans* retinol) to 11-*cis* retinaldehyde, delivery of that retinoid to the photoreceptors to generate rhodopsin (the rod-specific visual pigment) and the cone visual pigments, and conversion of the all-*trans* retinal hydrolyzed from the visual pigment protein upon absorption of light (thus initiating the visual phototransduction process) back to 11-*cis* retinaldehyde. In vertebrates, this also entails several other steps as well as a series of enzymes, binding partners, and transport proteins; the latter are required to shuttle the lipid-soluble retinoids through the aqueous extracellular environment. The neighboring retinal pigment epithelium (RPE) as well as Müller glial cells in the neural retina play parallel roles in this cycle, regenerating 11-*cis* retinal and supplying it to rods and cones, respectively. Choi *et al*. (1) describe the essential elements of the visual cycle, comparing its operation in vertebrates versus invertebrates. In particular, they highlight the central role of RGR, the RPE-retinal G protein-coupled receptor, as the mammalian retinaldehyde photoisomerase, which acts along with RPE65 (retinoid isomerase) to provide a continuous supply of 11-*cis* retinaldehyde to the photoreceptors, both in darkness as well as under a range of light intensities.

Not all of the all-*trans* retinaldehyde liberated from the visual pigments upon photon absorption goes back into the visual cycle. Some of those molecules partition into the disc membranes of the photoreceptor “outer segment,” where they form a Schiff base conjugate with phosphatidylethanolamine (PE) to form N-retinylidene-PE (NRPE). This pool of molecules can serve as a transient membrane-bound depot of retinoid that, upon hydrolysis, can be released and re-enter the visual cycle; alternatively, a second molecule of retinaldehyde can covalently bind to NRPE to form a family of molecules known as “bisretinoids.” These molecules are fluorescent as well as potentially cytotoxic. Kim and Sparrow (2) provide an overview of bisretinoid metabolism, biology, and known retinal disease associations.

Xanthophylls, such as lutein and zeaxanthin, are a subclass of the larger family of lipid-soluble natural products known as carotenoids. They are plant-derived dietary constituents that become concentrated in the central zone of the retina known as the fovea in humans and nonhuman primates; there, they comprise the macular pigment that gives the fovea its characteristic yellow-orange color. Bernstein and Arunkumar (3) review the chemistry, metabolism, and physiological function of this class of molecules in the retina. Of importance, they provide an overview of the role of lutein and zeaxanthin across the lifespan in humans, from early pre- and postnatal ocular development throughout the aging process. They also discuss the relationship between xanthophyll status and human eye diseases at both ends of the lifespan, including retinopathy of prematurity (ROP) as well as age-related macular degeneration (AMD), and speculate on the potential therapeutic value of dietary xanthophyll supplementation to prevent or reduce the severity of such diseases.

Fatty acids are a diverse class of ubiquitous lipids found in all cell types and tissues and exist either in the free (unesterified) form or covalently incorporated into other lipid classes, such as glycerophospholipids, triglycerides, sphingolipids, and steryl esters. Fu *et al*. (4) present evidence supporting the case for fatty acid oxidation as a significant source of energy (ATP) serving the metabolic needs, viability, and function of retinal photoreceptor cells (the rods and cones). For decades, it generally has been assumed that glucose is the primary carbon source used for ATP production by the mitochondria of photoreceptor cells, as it is in the brain. The cellular compartment known as the ellipsoid of the photoreceptor cell's “inner segment” (cell body) is densely packed with mitochondria; these cells have extremely high energy demands and are able to “burn” fatty acids as fuel via oxidative phosphorylation to generate ATP, much like skeletal and cardiac muscle cells. Exactly which fatty acids are preferentially utilized in vivo for ATP production is still a matter of speculation. There also is an important interplay between the photoreceptors of the neural retina and the neighboring RPE cells. This monolayer of stationary phagocytic cells forms the interface between the neural retina and the choroidal blood supply, the latter being the primary source of nutrients (including glucose and lipids) for the photoreceptor cells. Alternative energy substrates and the contributions of other cells, particularly Müller glia, to overall energy balance in photoreceptors is discussed as well. In addition, the authors open the door to modulation of fatty acid oxidation as a possible therapeutic intervention for retinal degenerative diseases that primarily impact photoreceptor function and viability.

The retina is highly enriched in polyunsaturated fatty acids, particularly docosahexaenoic acid (DHA; C22:6,n-3), which comprises >50% of the total fatty composition of vertebrate photoreceptor outer segment membranes. Although DHA exists primarily esterified to glycerophospholipids in the neural retina and RPE, a portion of the overall DHA pool serves as the essential substrate to form “docosanoids,” such as the neuroprotectins and resolvins (see also discussion below). In addition, DHA, eicosapentaenoic acid (C20:5,n-3), and arachidonic acid (C20:4,n-6) undergo elongation by the fatty acid elongase ELOVL4 (elongation of very long chain fatty acids protein 4), resulting in the formation of *omega*-3 and *omega*-6 very long-chain polyunsaturated fatty acids (VLC-PUFAs). In the retina, these fatty acids are primarily incorporated into phosphatidylcholine lipid species. Up until recently, the biological significance of these molecules was not known. However, that mystery has been solved by the discovery of a novel class of molecules called “elovanoids,” so named because their formation is catalyzed by ELOVL4. In the RPE, elovanoids are derived from 32- and 34-carbon *omega*-3 VLC-PUFAs. Nicolas Bazan (5) reviews the discovery of elovanoids, providing a perspective on their chemistry and their bioactivities that target fundamental prohomeostatic and neuroprotective signaling mechanisms in the RPE cell. These novel lipid mediators downregulate senescence gene programming, SASP (senescence-associated secretory phenotype), and inflammaging (chronic, age-dependent, low-grade inflammation); in turn, this promotes and sustains photoreceptor cell structural integrity, viability, and function. The subject of ELOVL4 and the formation of VLC-fatty acids, including saturated (VLC-SFAs) and polyunsaturated (VLC-PUFAs) species, is amplified in the review article by Yeboah *et al*. (6). They provide an historical perspective on the discovery of ELOVL4 and describe how mutations in this enzyme result in a spectrum of profoundly severe diseases with very distinct phenotypes, including those affecting the eye (e.g., early-onset macular dystrophy) and the brain (e.g., age-related cerebellar ataxia and atrophy). The prospects for development of novel classes of therapeutics to intervene in these blinding and neurological disorders are also discussed.

Sterols represent yet another class of quantitatively significant and biologically important molecules in the retina as well as in other ocular tissues. Cholesterol represents >98% of the total sterols found in vertebrate retinas, under normal conditions. By comparison, cholesteryl esters represent a relatively minor fraction of the overall sterol pool, primarily serving as a transient storage depot or transport form of cholesterol. Ramachandra Rao and Fliesler (7) review the topic of cholesterol homeostasis in the retina, both under normal as well as pathological conditions, considering the relative contributions of local de novo synthesis versus uptake from the blood as well as intraretinal exchange of cholesterol and cholesteryl esters, e.g., between the RPE and photoreceptors or between the Müller glia and photoreceptors. They also discuss animal models of human hereditary diseases involving defects in cholesterol biosynthesis (e.g., Smith-Lemli-Opitz syndrome) leading to retinal degeneration and the potential involvement of oxysterols in such diseases. They conclude with a perspective on the remaining unanswered questions regarding cholesterol synthesis, import/export, distribution in the different cell types and cellular layers of the retina, and emerging tools and technologies that might be utilized to answer these questions.

Phosphoinositides (PIs) are a quantitatively minor, yet biologically important class of lipids that are intricately involved in a variety of cellular signaling processes in the retina as well as in the CNS in general and other tissues throughout the body. Beyond this, PIs have been implicated in a broad spectrum of cellular processes, including ciliogenesis, protein trafficking, vesicular transport, phagocytosis, autophagy, and synaptic functions. Rajala (8) provides a review of the history of PI discovery, the fundamental features of PI chemistry, biochemistry and enzymology, the involvement of PI in signaling pathways both in the vertebrate as well as invertebrate retina, methods for detecting PI species, and the link between dysfunctional PI metabolism and retinal degeneration.

Sphingolipids serve a myriad of functions in ocular tissues. Simon *et al*. (9) provide a broad overview of these functions with a focus on the role of sphingolipids in the normal physiology of the retina, as well as their involvement in retinal diseases. They first give a brief historical perspective regarding sphingolipid discovery, followed by a discussion of sphingolipid chemistry and metabolism and the critical role sphingolipids play in determining cell survival versus cell death. They discuss involvement of sphingolipids in a wide variety of ocular pathologies, including age-related macular degeneration (AMD), uveitis (neuroinflammation of the retina), glaucoma, retinitis pigmentosa, vitelliform macular dystrophy (*a.k.a.* Best disease), macular telangiectasia, and diabetic retinopathy. They conclude with a discussion of some remaining questions regarding the distribution of sphingolipids and their associated enzymes among the various cell types and cellular layers of the retina, and the further delineation of their roles in the retina in health and disease.

Diabetic retinopathy (DR) is among the top three causes of progressive, irreversible blindness worldwide. Busik (10) provides a succinct overview of defects in lipid metabolism as they relate specifically to DR. She discusses the outcomes of several large clinical trials regarding diabetes and the onset and progression of DR, as well as the ameliorative impact of lipid-lowering drugs (e.g., fibrates, statins) and dietary lipids, particular *omega*-3 fatty acid supplementation. The specific involvement of fatty acid, cholesterol, and sphingolipid (especially ceramide) metabolism, as well as activation of PPARα, in DR is also discussed.

The ocular lens is an unusual tissue from several standpoints. First, the overwhelming majority of the cells that comprise the lens (known as lens fiber cells) have no internal organelles; they consist of a plasma membrane encapsulating cytoplasm, the latter being highly enriched in a family of small, soluble proteins called “crystallins.” Second, rather than being replaced as they age, the fiber cells become compacted progressively into the interior region of the lens, where they remain throughout life, and the lipids and proteins that comprise the lens fiber cell plasma membrane do not turn over. Third, the lens is completely avascular, relying exclusively on diffusion of nutrients present in the aqueous humor at its anterior side and the vitreous humor at its posterior side. Fourth, the predominant lipid constituent of the lens is dihydrosphingomyelin, which is not found in appreciable amounts in other tissues or cells; also, the cholesterol-to-phospholipid molar ratio of lens membranes is as high as 10:1, almost the reverse of what that ratio is in most other tissues and cells. Normally, the lens is transparent, allowing the light that enters the eye through the pupil to reach the retina, while also helping to focus that light (aided by the refractive power of the cornea) so as to create a sharp image on the retina. However, various genetic as well as environmental, traumatic, and age-dependent processes can result in lens opacification (cataract formation), thus distorting and compromising vision. Borchman (11) reviews the structure, composition, and function of lens cell membranes and the factors that contribute to the formation of cataracts. He also describes the composition, formation, structure, and function of the tear film lipid layer (TFLL), which comprises the surface layer of the aqueous tear fluid that bathes the anterior surface of the cornea, keeping the cornea hydrated and optically clear. The TFLL also contains some unusual lipid species, not found in the lens or most other tissues of the body, wax esters and cholesteryl esters with unusually long hydrocarbon chains, which help to maintain TFLL integrity. Instability of the TFLL contributes to the derangement of the tear film spreading between blinks, resulting in a condition known as dry eye disease. Borchman's review focuses on the use of biophysical tools, particularly spectroscopy, to define the relationship between lipid conformational order and the origins of cataract formation and dry eye disease.

Finally, Pham and Haydee Bazan (12) review the biological actions of docosanoid-dependent signaling in the context of corneal nerve regeneration and sequelae relevant to tear formation, DED, corneal wound repair, and neuropathic pain. They discuss the evidence that links the release of DHA from glycerophospholipids (stimulated by a specific phospholipase A2) to the formation of neuroprotectin D1 (NPD1) and the novel resolvin RvD6i, which in turn stimulates the formation of growth factors (BDNF, NGF) and semiphorin 7A to promote corneal nerve regeneration, wound healing, and tear formation. RvD6i also modulates gene expression in the trigeminal ganglia (the sensory nerves of which innervate the cornea), which promotes neurogenesis and reduces the neuropathic pain associated with diseases and trauma that cause corneal wounds, ulceration, and nerve damage. The translational implications of these findings for the development of new, effective treatments for such diseases and trauma-induced conditions are discussed.

Although the roles played by various lipids and lipid-soluble molecules in the eye have been delineated in considerable detail over the years with regard to both normal and pathological physiology, we still are far from a complete understanding of this aspect of biology. As ongoing research in this arena sheds new light on fundamental biological processes at work in the various neuronal and nonneuronal cells and tissues of the eye, and the means to regulate them, those insights hold great promise for major advancements in developing new treatments and cures for diseases that result in compromised vision and progressive blindness.

## Author ORCIDs

Steven J. Fliesler https://orcid.org/0000-0002-2557-142X
